# Effect of participation in a clinical trial on glycemic control in type 2 diabetic patients

**DOI:** 10.4103/0253-7613.68442

**Published:** 2010-08

**Authors:** Mukta N. Chowta, Prabha M. Adhikari, Nithyananda K. Chowta, Susan D’Souza, Ashok K. Shenoy

**Affiliations:** Departments of Pharmacology and Medicine, Kasturba Medical College, Mangalore, Manipal University, Karnataka, India

Sir,

Improved self-management following participation in a clinical trial with better glycemic control has been recently reported in the literature.[1,2] Participation in a clinical trial encourages a greater involvement of patient in self-care. It also results in more equal partnership between patients and health care professionals. The present retrospective analyses aims at examining the influence of study participation on glycemic control in type 2 diabetic patients.

Retrospective analysis of glycemic control in type 2 diabetic patients who participated in clinical trials was done by comparing the fasting glucose and HbA1C values at screening and randomization visits. We selected the patients who participated in a randomized controlled clinical trial on new therapy for type 2 diabetes mellitus. A total of three such studies were selected. All these studies were approved by the Institutional Ethics Committee. Eligible patients for the evaluation were those who undertook both the screening as well as randomization visits. Those who had undergone only screening were excluded. The interval between the two visits varied between studies depending upon the study design. Antidiabetic medication remained unchanged between the two visits. Fasting glucose, HbA1C, and body weight were measured at both the visits. All these trials included adult, males or females.

Trial 1 included obese patients with features of metabolic syndrome (body mass index above 25 kg/m^2^) who were inadequately controlled with insulin alone or in combination with oral hypoglycemic agents. Patients were screened at visit 1 and those found eligible continued with stable dose of insulin and oral hypoglycemic agents along with placebo medication for 2 months. At the end of 2 months patients were randomized to receive active study medication. Trial 2 included type 2 diabetic patients with inadequate glycemic control. Patients were drug naïve at the time of inclusion. Patients were screened at visit 1 and received placebo for 1 month after which the eligible patients were randomized. Trial 3 included patients with history of type 2 diabetes mellitus and body mass index between 23 and 45 kg/m^2^. These were inadequately controlled (HbA1C above 8.0%) and received a combination therapy including metformin and another oral hypoglycemic agent. After screening, all eligible patients were put on maximum tolerated dose of metformin along with pioglitazone for 3 months after which the eligible patients were randomized to receive the study medication.

Fasting glucose and HbA1C were measured at screening as well as randomization visit in all the three studies. Glucometer was dispensed to all patients for self-monitoring of blood glucose. Patients were given adequate education and were taught to use the glucometer. They were also advised to maintain a diary for daily record of glucometer readings. Data from all three studies were combined as well as analyzed individually. The Student ‘t’-test was used for statistical analysis and a *P* < 0.05 was considered significant. The main outcome was short-term glycemic control measured by glycated hemoglobin (%) and fasting glucose level.

A total of 61 patients (32 males and 29 females) were studied (trial l: 17, trial 2: 25 and trial 3: 19). Patients in trial 3 had maximum duration of diabetes (17.65±8.02 years) and trial 2 patients had shortest. The median interval between screening (visit 1) and randomization (visit 2) was 60 days in trial 1, 30 days in trial 2, and 90 days in trial 3.

Baseline HbA1C was highest in trial 1 patients. It increased further in trial 1 patients from baseline to randomization visit, whereas it decreased in trial 2 and 3 patients. Drop in HbA1C was much more in trial 3 patients though statistically insignificant (*P*=0.074). Overall analysis has shown a decrease in HbA1C between the two visits. Baseline fasting blood glucose was also highest in trial 1 patients and was lowest in trial 2 patients. Fasting blood glucose increased from visit 1 to visit 2 in trial 1 patients and decreased in trial 2 and 3 patients. The drop in fasting glucose level in trial 3 patients was statistically significant (*P*=0.01). Overall analysis also showed a decrease in fasting glucose level between the two visits [Table 1]. Body weight increased from screening to randomization visit in all the trials except trial 2 where a small decrease was noted. Overall analysis showed an increase in body weight between the two visits. A mean increase in body weight in trial 3 patients was statistically significant.

**Table 1:** Fasting blood glucose (mmol/L) and Hba1C (%) at screening and randomization visits

*Group*		*Visit 1*	*Visit 2*	*Mean change*	*Median interval (days)*	*P value*
Trial 1	FBS	9.95 ± 3.85	10.86 ± 3.07	0.04 ± 3.05	60	0.075
	Hba1C	9.28 ± 1.52	9.48 ± 1.19	0.14 ± 1.13	60	0.103
Trial 2	FBS	8.22 ± 2.17	7.38 ± 1.33	-0.60 ± 2.8	30	0.201
	Hba1C	7.81 ± 0.7	7.86 ± 0.69	0.09 ± 0.58	30	0.262
Trial 3	FBS	9.22 ± 2.22	7.81 ± 1.89	-0.37 ± 0.74	90	0.01*
	Hba1C	9.22 ± 1.36	8.87 ±1.28	-0.37 ± 0.74	90	0.074
Overall	FBS	9.13 ± 2.87	8.75 ± 2.67	-0.04 ± 3.05	30-90	0.47
	Hba1C	8.66 ± 1.37	8.52 ± 1.5	-0.14 ± 1.13	30-90	0.203

Values are expressed as mean ± SD;

* Highly significant (Student ‘t’-test).

The present analysis has shown that study participation itself causes sufficient changes in the glycemic control. Combined analysis of all the three studies has shown that there was sufficient reduction in blood glucose and HbA1c level in patients who participated in clinical trials. Similar observations have also been made before.[2,3] In the present study, patients who were in trial 1 have shown poorer glycemic control when compared to other two trials. Poor baseline glycemic control as well as long duration of diabetes could be the reason for this observation. In contrast, Gale *et al*.[3] have reported that reduction in blood sugar concentration is proportional to initial HbA1C with large decreases in those with the poor initial control but no overall change in those at or below the 10^th^ percentile of HbA1C. Improved glycemic control following participation in a clinical trial can be best understood as a result of enhanced awareness in patients, increased care given by health professionals, frequent monitoring and motivation of patients for better adherence to therapy and lifestyle modification.[4,5] It is observed that people tend to alter their behavior when they know that they are being studied.[3] Our results also imply that the benefits seen in uncontrolled trials should be interpreted with extreme caution. It is advisable to have a run-in or lead-in phase of adequate length before randomization in a clinical trial to achieve a stable baseline value of HbA1c.
